# Condition-specific or generic preference-based measures in oncology? A comparison of the EORTC-8D and the EQ-5D-3L

**DOI:** 10.1007/s11136-016-1443-y

**Published:** 2016-11-09

**Authors:** Paula K. Lorgelly, Brett Doble, Donna Rowen, John Brazier, Cancer 2015 investigators, David M Thomas, Stephen B Fox, Heather Thorne, John P Parisot, Ken Doig, Andrew Fellowes, Alexander Dobrovic, Paul A James, Lara Lipton, David Ashley, Theresa Hayes, Paul McMurrick, Gary Richardson, Paula Lorgelly, Mark Lucas, John J McNeil, Tom John

**Affiliations:** 1 Office of Health Economics, 7th Floor, 105 Victoria Street, London, SW1E 6QT UK; 2 Faculty of Business and Economics, Centre for Health Economics, Monash University, Clayton, VIC Australia; 3 Cambridge Centre for Health Services Research, University of Cambridge, Cambridge, UK; 4 School of Health and Related Research (ScHARR), University of Sheffield, Sheffield, UK

**Keywords:** Cancer, Condition-specific non-preference-based measures, Generic preference-based measures, Quality of life

## Abstract

**Purpose:**

It has been argued that generic health-related quality of life measures are not sensitive to certain disease-specific improvements; condition-specific preference-based measures may offer a better alternative. This paper assesses the validity, responsiveness and sensitivity of a cancer-specific preference-based measure, the EORTC-8D, relative to the EQ-5D-3L.

**Methods:**

A longitudinal prospective population-based cancer genomic cohort, Cancer 2015, was utilised in the analysis. EQ-5D-3L and the EORTC QLQ-C30 (which gives EORTC-8D values) were asked at baseline (diagnosis) and at various follow-up points (3 months, 6 months, 12 months). Baseline values were assessed for convergent validity, ceiling effects, agreement and sensitivity. Quality-adjusted life-years (QALYs) were estimated and similarly assessed. Multivariate regression analyses were employed to understand the determinants of the difference in QALYs.

**Results:**

Complete case analysis of 1678 patients found that the EQ-5D-3L values at baseline were significantly lower than the EORTC-8D values (0.748 vs 0.829, *p* < 0.001). While the correlation between the instruments was high, agreement between the instruments was poor. The baseline health state values using both instruments were found to be sensitive to a number of patient and disease characteristics, and discrimination between disease states was found to be similar. Mean generic QALYs (estimated using the EQ-5D-3L) were significantly lower than condition-specific QALYs (estimated using the EORTC-8D) (0.860 vs 0.909, *p* < 0.001). The discriminatory power of both QALYs was similar.

**Conclusions:**

When comparing a generic and condition-specific preference-based instrument, divergences are apparent in both baseline health state values and in the estimated QALYs over time for cancer patients. The variability in sensitivity between the baseline values and the QALY estimations means researchers and decision makers are advised to be cautious if using the instruments interchangeably.

## Introduction

Cost-utility analyses (CUA) require preference-based measures (PBMs) of outcome. Traditionally PBMs, so-called multi-attribute utility instruments (MAUIs), have been generic. The mostly commonly employed generic PBM is the EQ-5D [1], a measure which the National Institute for Health and Care Excellence (NICE) actively encourages [2]. While the use of the same measure across a range of diseases and conditions increases comparability (what NICE refers to as a need for consistency) when informing decisions, there have been criticisms that these generic measures are not sensitive to certain disease-specific characteristics [3–5]. This is not withstanding the fact that such PBMs have been found to be sensitive to health issues that the instrument does not explicitly ask about. For example, the EQ-5D has been found to be sensitive to a range of clinical features in patients with Parkinson’s disease including hallucinations [6]. Sensitivity is therefore a grey area. While using PBMs in some diseases may mean that important clinical and patient quality of life changes are missed entirely, in other disease areas it may be that effects are found, but the magnitude of these is underestimated. That is, it is not a simple question of whether PBMs are sensitive, but whether they are sensitive enough?

There are a number of ways in which health economists can introduce disease-specific sensitivity to the assessment of outcomes in a CUA [7]. Often they utilise mapping algorithms which estimate the relationship between a condition-specific non-preference-based measure and a generic PBM [8, 9]. A more resource intensive alternative is to elicit preferences from patients (or the general public) for condition-specific vignettes describing a health state, that is to use preference elicitation techniques like time trade-off, standard gamble or a discrete choice experiment within the study population [10, 11]. A third alternative is the use of bolt-ons to existing generic instruments, like adding vision impairment or hearing impairment to the EQ-5D [12]. Bolt-ons are thought to improve a generic instrument’s content validity for a particular condition, but also retain a core instrument that is comparable across conditions. A further option which is growing in popularity is to develop condition-specific preference-based measures (CSPBMs). CSPBMs can be developed from first principles (determine dimensions that are important to a patient/sufferer, design the instrument, undertake a valuation study and produce a set of tariffs) [13], or one could modify (in many instances this means reduce) an existing non-preference-based measure and undertake a valuation study [14]. As these non-preference-based measures have already been developed for the condition, arguably they have already been assessed for validity and sensitivity. An additional benefit of using existing non-preference-based measures is that clinicians can get information on quality of life and disease dimensions of interest to them, while health economists are able to estimate health state values for use in a CUA without the need to administer an additional outcome measure.

Despite the apparent benefits of CSPBMs, their use is limited. If CSPBMs are to be more widely adopted, then evidence of their performance is required. This does not negate that many health technology appraisal (HTA) agencies have an explicit preference for generic instruments, so it may be that even in the face of compelling evidence of the benefits of CSPBMs implementation will be restrained. Leaving this debate aside (interested readers are referred to Versteegh et al. [5] and Brazier and Tsuchiya [15]), this paper—using oncology as a case study—seeks to assess the validity, responsiveness and sensitivity of a cancer-specific preference-based measure, the EORTC-8D, relative to a generic PBM, the EQ-5D-3L.

## Methods

### Data

Cancer 2015 is a large-scale prospective longitudinal population-based molecular cohort study [16]. It enrols newly diagnosed/treatment naïve cancer patients irrespective of the tumour site (except leukaemia) and at all stages of disease. Recruitment is staged, and phase 1 (2011–2014) targeted the enrolment of 1000 patients from five hospitals in Victoria, Australia. It aims to test and implement a new model of cancer diagnosis and treatment with a specific focus on integrating molecular pathology into routine cancer diagnosis [17], whereby all tumours are genotyped and actionable mutations identified so to inform cancer diagnosis, prognosis and treatment at an individual level. The new model is one where personalised treatment plans, specifically precision medicines guided by genomic testing, would be offered to patients.

Patients consent to have their tumour biopsy and blood screened using next-generation sequencing (NGS) [18]. A baseline questionnaire collects information on patient socio-demographics and patient and familial history. Clinical records including pathology results are drawn upon to gather information on tumour site and stage and treatment intentions (including changing intentions over time). Patients are also asked to complete three patient-reported outcome measures (PROMs); see “Instruments” section for further details. PROMs are repeated at 6- and 12-month follow-up (for those with advanced disease the first follow-up point was at 3 months).

### Instruments

The European Organization for Research and Treatment of Cancer Quality of Life Questionnaire C30 (EORTC QLQ-C30) is a non-preference-based health-related quality of life (HRQoL) measure which is frequently employed in cancer clinical trials. It is one of a suite of EORTC instruments and the C30 is regarded as a ‘generic’ cancer measure (e.g. EORTC QLQ-BR23 is specific to breast cancer, while EORTC QLQ-MY20 is for myeloma) [19]. It includes 30 questions which feed into nine multi-item scales: five functional scales (physical, role, cognitive, emotional and social functioning); three symptom scales (fatigue, pain, and nausea and vomiting); and a global health status/quality of life scale. Six single-item scales mainly for symptoms are also included (dyspnoea, insomnia, appetite loss, constipation, diarrhoea and financial difficulties). Cancer 2015 included version 3.0, the most recent version, which is recommended by the EORTC Quality of Life Group. The instrument is scored so that it provides summary scores (between 0 and 100) for a patient’s functioning, symptoms and global quality of life, where a higher score represents a higher (‘better’) level of functioning or quality of life or a higher (‘worse’) level of symptoms.

The EORTC-8D has eight dimensions (physical functioning, role functioning, pain, emotional functioning, social functioning, nausea, fatigue and sleep disturbance, and constipation and diarrhoea) each with 4 levels except physical functioning which has 5 levels. The instrument was derived using Rasch and factor analysis from the EORTC QLQ-C30 [20] with the EORTC-8D drawing on 10 questions from a possible 30. There are 81,920 unique health states in the EORTC-8D which were valued using a time trade-off approach in a sample of the general population from the north of England. The resulting values range from 0.292 to 1.00, on the full health–dead 1–0 scale.

The EQ-5D-3L (previous known as the EQ-5D) has five dimensions (mobility, self-care, usual activities, pain/discomfort and anxiety/depression) each with three levels such that there are 243 health states [21]. The EQ-5D-3L was also valued using a time trade-off approach in the UK. Other country valuations exist, some of which use other valuation techniques (including a discrete choice experiment (DCE) in Australia [22]); however, this analysis ignores any cross-country differences, and undertakes the comparison using UK tariffs employing the UK tariff (MVH-A1 algorithm) for the EQ-5D-3L [23]. The scoring range for the EQ-5D-3L in the UK is from −0.594 to 1.00, i.e. it includes states that are worse than dead (<0).

Cancer 2015 also included the EORTC-8D as an instrument in its own right (i.e. both the 30-item EORTC QLQ-C30 and the 10-item EORTC-8D were administered meaning that 10 items were duplicated in the questionnaire), in contrast to deriving EORTC-8D values indirectly from EORTC QLQ-C30 responses. Although the EORTC-8D has not been validated (e.g. psychometrically assessed) as a standalone instrument, it was deemed interesting to compare the responses of the standalone EORTC-8D with the derived EORTC-8D given that the standalone instrument is shorter (10 vs 30 questions). Our analysis ignores the responses to the standalone instrument, however, and only utilises the derived responses as they were found to be highly correlated (*r* = 0.93) and the standalone EORTC-8D instrument, despite being shorter, had more missing responses (which was possibly due to an ordering effect in the questionnaire because the standalone instrument was included after the C30). Note that phase 2 of Cancer 2015 does not include the EORTC-8D as a standalone instrument.

### Analysis

Using the baseline health state values, we assessed the normalities of the distribution of each instrument using both the Shapiro–Wilk W test and the Shapiro–Francia W test. Skewness and kurtosis were also assessed.

We then assessed the correlation both within dimensions (using Spearman’s rank) and the health state values as a whole (using Pearson’s R). We used this correlation matrix analysis to consider convergent validity (i.e. the degree to which an instrument/dimension correlates with another instrument/dimension measuring the same concept) [24]. We expect there to be convergent validity in the items which are similar, e.g. those measuring physical dimensions of health and those measuring psychological dimensions. Strength of correlation was based on the following thresholds: *r* = 0–0.2 (very weak), *r* = 0.2–0.4 (weak), *r* = 0.4–0.7 (moderate), *r* = 0.7–0.9 (strong), *r* = 0.9–1.0 (very strong) [25]. It is important to note that the EQ-5D does not claim to perform *measurement* within its dimensions (i.e. it does not measure mobility), but instead provides a simple classification system; however, correlations within dimensions are commonly undertaken in assessments of validity [26, 27]. We additionally explored ceiling effects in each instrument by considering the relationship between item dimensions in one instrument and full health in the other instrument as measured by a health state value of 1, e.g. EORTC-8D item responses when the EQ-5D-3L is one and vice versa.

Agreement between the instruments was examined using a Bland–Altman plot [28]. This plotted the difference between EORTC-8D and EQ-5D-3L values against the mean of the values for each individual. The mean difference provides the estimate of bias while the limits of agreement, LOA (based on a ±1.96 × SD_difference_ interval), provide an estimate of the influence of random variation. If there was good agreement between the EORTC-8D and the EQ-5D-3L, then only 5% of points would lie outside of the LOA. Agreement was further assessed by estimating the intra-class correlation coefficient (ICC) [29] (two way mixed effects with absolute agreement). Strength of agreement was based on the following thresholds: ICC = 0–0.2 (poor), ICC = 0.2–0.4 (fair), ICC = 0.4–0.6 (moderate), ICC = 0.6–0.8 (strong) and ICC > 0.8 (almost perfect) [29, 30].

To understand the construct validity of each measure, that is whether the instrument is sensitive (or indeed more sensitive) to different covariates [24], we compared mean health state values using paired t tests and ANOVAs where appropriate, and estimated the standardised effect size (difference in means divided by the standard deviation). The covariates included age, gender, site of recruitment, insurance status, smoking status, performance/functioning status (measured using the Eastern Oncology Cooperative Group (ECOG) performance status scale) at baseline and over time, initial treatment intention (as an indicator for severity: none, curative, palliative), planned initial follow-up point (again as an indicator for severity), status at follow-up (dead or alive), site of tumour and staging of the disease. We hypothesised that the EORTC-8D would have a greater ability to discriminate between the disease characteristics (functioning, severity, stage) than the EQ-5D-3L. We additionally hypothesised that for the patient-level characteristics (age, gender, insurance status, etc.) both instruments should have similar levels of discriminatory power as they are unrelated to condition.

QALYs were estimated using the area under the curve method. The average time of follow-up was 434 days. Those who died were given a health state value of zero at their date of death and included in the QALY calculation. Correlation between the generic QALYs and condition-specific QALYs was assessed using Pearson’s R correlation coefficient. The sensitivity of the QALY estimates to various covariates (as described above) was also explored in bivariate analyses in order to further assess construct validity. As above, we hypothesise that there will be more discrimination with the condition-specific QALYs than with the generic QALYs for the covariates which reflect disease characteristics, but they will have equal discriminatory power for the patient-level characteristics. Regression analysis was employed to further examine the extent to which the difference in QALYs (condition-specific minus generic QALYs) was influenced by baseline patient demographics, disease characteristics, indicators of severity, change in patient’s performance/functioning (ECOG) status overtime and the difference in baseline health state values. A linear model was imposed and the regression was multivariate with all variables included at the same time.

All statistical analyses were undertaken in STATA MP version 13.0.

## Results

Cancer 2015 recruited its first patient in November 2011, and as of February 2015 there were 1829 patients enrolled in the cohort; however, not all patients have complete PROMs data. We have baseline EQ-5D-3L values for 1715 patients and EORTC-8D values for 1689 patients, and the complete case sample (where there is a baseline value for both instruments) is 1678. We are able to estimate generic and condition-specific QALYs for 1157 patients. Note that 269 patients (nearly 15% of those recruited) have died.

Table 1 presents the descriptive statistics for the sample at baseline. The sample is elderly (mean age 62), the majority are male (54%), and a large number have other co-morbidities as measured by the Charlson Comorbidity Index (mostly diabetes and arthritis) [31]. The cohort purposely included a private hospital in the sample (in order to make treatment comparisons at a later date, and also because Australia has a two-tiered health care system); this hospital contributed 19% of the patients, but 43% of the total sample have insurance cover for hospitals. The hospital insurance variable can be considered to be reflective of income, as at a certain income threshold private health insurance is incentivised (i.e. an additional tax is imposed on high income earners who do not have insurance).

**Table 1:** Baseline sample descriptive statistics

	Mean (range) or percentage	*N*
Age at consent	61.7 (18, 92)	1678
Charlson Comorbidity Index	2.24 (0, 14)	1510
Gender		
Female	45.8%	768
Male	54.2%	909
Recruiting hospital		
Public	81.2%	1362
Private	18.8%	316
Hospital insurance cover		
Yes	42.5%	697
No	57.5%	943
Smoking status		
Current smoker	14.5%	235
Ex-smoker	46.7%	756
Never smoked	38.8%	628
Place of residence		
Major city	46.4%	776
Inner regional	47.0%	787
Outer regional	6.6%	111
Tumour site		
Prostate	14.9%	249
Breast	19.8%	332
Head and neck	13.2%	221
Colorectal	10.8%	180
Lung	10.3%	172
Bone and soft tissue	3.1%	52
Cervical	2.7%	45
Cancer unknown primary	2.7%	45
Renal	3.5%	58
Oesophagogastric	4.0%	67
Other (includes 12 known sites)	15.2%	254
Stage^a^		
Stage 0	0.9%	15
Stage 1	20.4%	343
Stage 2	23.5%	394
Stage 3	17.8%	298
Stage 4	16.8%	282
Other	6.2%	104
Stage not available	14.4%	242
Treatment intentions		
No treatment	1.5%	23
Curative	82.2%	1262
Palliative	16.3%	251
ECOG^b^ score		
Normal activity	66.6%	1086
Limited in normal activity	23.4%	381
Self-care capable but not working	7.4%	120
Limited self-care	2.5%	40
No self-care	0.2%	3

^a^Stage 0 ‘*in situ*’ cancer, Stage 1 localised cancer, Stage 2 regional spread in the general region it first began including nearest lymph nodes, Stage 3 regional spread and more extensive lymph node involvement, Stage 4 distant spread, and Other that could not be classified into any other stage

^b^ *ECOG* Eastern Oncology Cooperative Group performance status scale

Note that the samples in each category do not always sum to 1678 as there are missing data

In terms of disease, breast cancer and prostate cancer contribute the most patients (20% and 15%, respectively) to the cohort, but there is representation across the spectrum of tumour sites. All stages of cancer (staged via the staging method appropriate to the tumour site) are represented, and the majority of patients are noted to have curative treatment intentions at enrolment (82%), although some patients have palliative treatment intentions. The large majority of patients (67%) have an ECOG performance status which aligns with normal activity [32].

### Baseline values

**Fig. 1:**
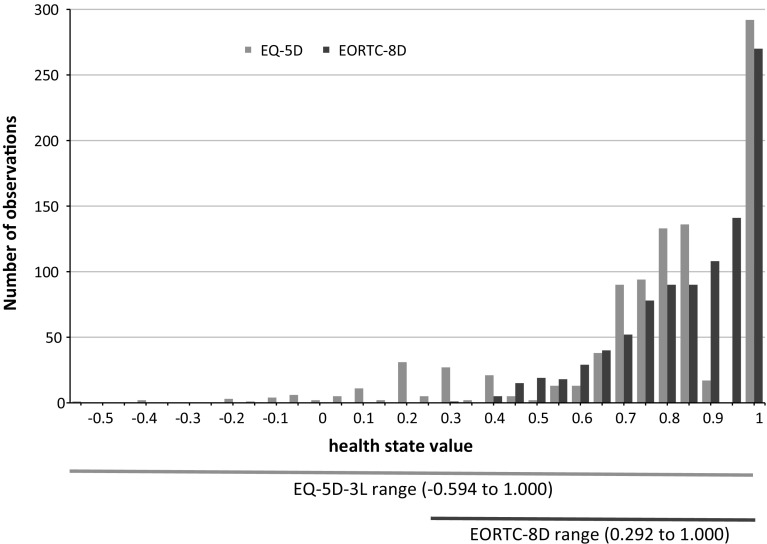
Histogram of baseline EQ-5D and EORTC-8D values

The EORTC QLQ-C30 summary measures are presented in Table 2; the mean functioning score was 79, mean symptom score was 19, and the mean global health score was 69. The mean EQ-5D-3L health state value was 0.748, while the mean health state value for the EORTC-8D was 0.829. The range of health state values is shown in Fig. 1 which plots the histograms for the baseline values for each instrument. The data are skewed and non-normal, and this is further supported in formal statistical tests (EQ-5D-3L Shapiro–Wilk test *z* = 11.8, *p* < 0.001; EORTC-8D Shapiro–Wilk test *z* = 10.1, *p* < 0.001; EQ-5D-3L Shapiro–Francia test *z* = 10.9, *p* < 0.001; EORTC-8D Shapiro–Francia test *z* = 9.5, *p* < 0.001).

**Table 2:** Descriptive statistics for health status

	Mean	SD	Min	Max	*N*
EQ-5D-3L at baseline	0.748	0.263	−0.594	1.000	1678
EORTC-8D at baseline	0.829	0.147	0.292	1.000	1678
EORTC QLQ-C30 functioning score at baseline	79.20	18.85	8.89	100	1656
EORTC QLQ-C30 symptom score at baseline	19.19	17.18	0	89.74	1655
EORTC QLQ-C30 global health score at baseline	69.00	23.37	0	100	1674
QALYs (from EQ-5D)	0.860	0.018	−0.108	3.138	1157
QALYs (from EORTC-8D)	0.909	0.018	0.001	3.078	1157

There is considerable variability across dimension responses in both instruments; see “Appendix”. The use of the highest level (no problems) in the EQ-5D-3L ranged from 91.6% of responses for usual activities to 48.3% of responses for pain/discomfort. For the EORTC-8D, the use of the highest level ranges from 77.8% of responses for nausea and 30.9% of responses for fatigue and sleep disturbance.

The convergent validity of the instruments is assessed by considering the correlations across the dimensions and the health state values. Table 3 shows that correlations between the dimensions of the EQ-5D-3L and the EORTC-8D are mostly moderate, particular in the dimensions which appear to be assessing similar constructs, e.g. physical functioning (EORTC-8D) and mobility (EQ-5D-3L), pain (EORTC-8D) and pain/discomfort (EQ-5D-3L), emotional functioning (EORTC-8D) and anxiety/depression (EQ-5D-3L). The correlation between the baseline health state values is 0.755, considered to be strong [25]. The correlations between the baseline health state values and the baseline EORTC QLQ-C30 summary scores are strong/very strong, ranging from 0.730 to 0.917, except for the correlation between the EQ-5D-3L health state value and the global quality of life summary score which is 0.651 (moderate).

**Table 3:** Correlations between health state dimensions at baseline

EORTC-8D	EQ-5D-3L				
	Mobility	Self-care	Usual activities	Pain/discomfort	Anxiety/depression
Physical functioning	0.599	0.349	0.537	0.436	0.213
Role functioning	0.388	0.352	0.643	0.441	0.243
Pain	0.416	0.289	0.549	0.622	0.261
Emotional functioning	0.171	0.137	0.258	0.265	0.634
Social functioning	0.354	0.341	0.586	0.410	0.314
Fatigue and sleep disturbance	0.331	0.278	0.490	0.439	0.340
Nausea	0.242	0.215	0.379	0.334	0.245
Constipation/diarrhoea	0.277	0.237	0.361	0.295	0.191

All correlations are significant at the *p* < 0.001 level

In the assessment of ceiling effects, Tables 4 and 5 show good content validity; when one instrument records a value of full health, this corresponds with the higher levels in each dimension in the other instrument. An exception to this is the fatigue and sleep disturbance dimension in the EORTC-8D; 40% of the responses are not at level 1 when their EQ-5D profiles suggest they are in full health. This suggests that the generic PBM, in this context, would fail to pick up impairments in fatigue and sleep disturbance.

**Table 4:** EORTC-8D responses when EQ-5D-3L = 1 (percentages)

	Level 1	Level 2	Level 3	Level 4	Level 5
Physical functioning	79.88	17.48	2.44	0.20	0.00
Role functioning	88.01	9.76	1.42	0.81	n/a
Pain	93.09	6.50	0.20	0.20	n/a
Emotional functioning	86.99	12.60	0.41	0.00	n/a
Social functioning	83.94	14.02	1.63	0.41	n/a
Fatigue and sleep disturbance	60.98	34.35	4.27	0.41	n/a
Nausea	94.72	5.08	0.20	0.00	n/a
Constipation/diarrhoea	80.69	16.46	2.03	0.81	n/a

**Table 5:** EQ-5D-3L responses when EORTC-8D = 1 (percentages)

	Level 1	Level 2	Level 3
Mobility	98.62	1.38	0.00
Self-care	100.00	0.00	0.00
Usual activities	98.17	1.36	0.46
Pain/discomfort	91.28	8.72	0.00
Anxiety/depression	92.66	6.88	0.46

**Fig. 2:**
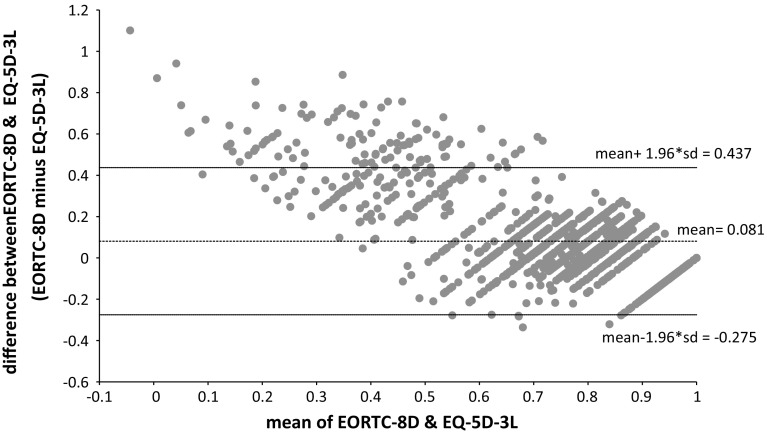
Bland–Altman plot of EORTC-8D and EQ-5D-3L at baseline

The ICC is 0.595 which suggests the agreement between the measures is moderate. The Bland–Altman plot in Fig. 2 suggests that there are small mean differences between the two instruments at baseline, but relatively wide limits of agreement. 6.97% of the data points are found to lie outside of the LOA suggesting poor agreement between the two measures, and this is particularly the case for the lower values of HRQoL.

An analysis of the sensitivity of each instrument to various subgroups including patient and disease characteristics (see Table 6, columns 1–4) finds that both the EQ-5D-3L and the EORTC-8D are sensitive to gender (females have lower baseline health state values), admission to hospital (public patients have lower health state values), smoking status (smokers, including ex-smokers, have lower health state values), stage of disease (metastatic cancer patients have lower health state values), hospital insurance (those without insurance have lower health state values), expected future follow-up (those with plans for follow-up at three months—i.e. more advanced disease—have lower baseline health state values) and ECOG score (those with worse scores have lower health state values). There is also variation in cancer site, and both instruments find that prostate cancer patients have the highest baseline health state values, while patients with lung cancer and cancer of the unknown primary (CUP) have the lowest baseline health state values. Although both instruments identify statistically significant differences within the covariates, it is notable that the variation in values is greater for the EQ-5D-3L. However, the standard deviation for the EORTC-8D is smaller, such that the estimated effect sizes (not shown) are larger for the EORTC-8D, although the differences in effect sizes between the EQ-5D-3L and the EORTC-8D are not significant. These findings imply that our initial hypothesis that the EORTC-8D would have greater discriminatory power with respect to the disease characteristics (functioning, severity, stage) is not refuted.

**Table 6:** Differences in baseline health state values and QALYs

	EQ-5D-3L baseline value		EORTC-8D baseline value		Generic QALYs		Condition-specific QALYs	
	Mean	*p* value	Mean	*p* value	Mean	*p* value	Mean	*p* value
Male	0.767	0.001	0.847	<0.001	0.889	0.079	0.940	0.059
Female	0.726		0.808		0.824		0.870	
Aged <30 years	0.722	0.307	0.792	0.005	0.751	<0.001	0.798	<0.001
Aged 30–50 years	0.724		0.818		0.943		1.003	
Aged 50–70 years	0.757		0.840		0.909		0.965	
Aged >70 years	0.743		0.814		0.714		0.742	
Public hospital recruitment	0.732	<0.001	0.819	<0.001	0.848	0.141	0.902	0.435
Private hospital recruitment	0.818		0.875		0.919		0.940	
Hospital insurance—no	0.717	<0.001	0.811	<0.001	0.822	0.006	0.876	0.017
Hospital insurance—yes	0.794		0.855		0.924		0.965	
Smoker	0.689	0.001	0.814	0.037	0.775	0.012	0.875	0.013
Ex-smoker	0.752		0.824		0.836		0.867	
Never smoked	0.763		0.840		0.930		0.982	
ECOG—normal activity	0.824	<0.001	0.878	<0.001	0.996	<0.001	1.037	<0.001
ECOG—limited in normal activity	0.671		0.771		0.715		0.775	
ECOG—self-care capable but not working	0.500		0.663		0.433		0.490	
ECOG—limited self-care	0.270		0.570		0.439		0.533	
ECOG—no self-care	0.190		0.689		0.020		0.030	
Change in ECOG—none	0.820	<0.001	0.871	<0.001	1.108	<0.001	1.147	<0.001
Change in ECOG—decline	0.675		0.783		0.495		0.558	
Change in ECOG—improvement	0.670		0.772		0.952		1.001	
Treatment intent—none	0.726	<0.001	0.818	<0.001	0.426	<0.001	0.601	<0.001
Treatment intent—curative	0.787		0.855		0.977		1.023	
Treatment intent—palliative	0.603		0.728		0.467		0.518	
Planned six month follow-up	0.762	<0.001	0.838	<0.001	0.934	<0.001	0.988	<0.001
Planned three month follow-up	0.631		0.757		0.420		0.441	
Alive at follow-up	0.772	<0.001	0.844	<0.001	1.002	<0.001	1.051	<0.001
Dead at follow-up	0.616		0.748		0.337		0.383	
Site—prostate	0.867	<0.001	0.921	<0.001	1.166	<0.001	1.220	<0.001
Site—breast	0.776		0.841		0.894		0.929	
Site—head and neck	0.718		0.843		0.910		0.996	
Site—colorectal	0.780		0.804		0.845		0.831	
Site—lung	0.647		0.758		0.600		0.652	
Site—bone and soft tissue	0.665		0.808		0.922		1.040	
Site—cervical	0.784		0.855		1.041		1.089	
Site—CUP	0.611		0.774		0.472		0.550	
Site—renal	0.735		0.813		0.755		0.770	
Site—oesophagogastric	0.686		0.789		0.570		0.610	
Site—all other	0.724		0.803		0.801		0.856	
Stage 0	0.750	<0.001	0.833	<0.001	0.756	<0.001	0.874	<0.001
Stage 1	0.781		0.849		1.056		1.102	
Stage 2	0.785		0.851		0.985		1.016	
Stage 3	0.784		0.846		0.901		0.947	
Stage 4	0.673		0.789		0.697		0.764	
Stage—other	0.657		0.782		0.596		0.663	
Stage—not staged	0.728		0.813		0.608		0.647	

### QALYs

**Fig. 3:**
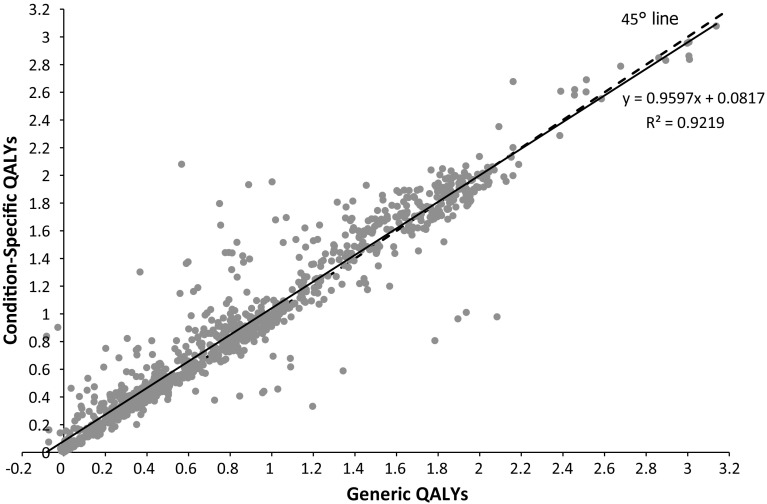
Correlation in QALY estimates

The estimated mean QALYs when using the EQ-5D-3L is 0.860 (range −0.108 to 3.138); the estimated mean QALYs when using the EORTC-8D is 0.909 (range 0.001–3.078); thus, the QALY estimates are higher for the condition-specific measure and the range is narrower (see Table 2). The difference while small (0.049) is statistically significant (*p* < 0.001, paired t test). The generic and condition-specific QALYs are very strongly correlated (Pearson’s *R* = 0.959); see Fig. 3.

The sensitivity of both types of QALYs to variations in the sample characteristics is presented in Table 6, columns 5–8. There are many similarities with the relationships that were found for baseline health state values (columns 1–4), although some significant relationships are no longer apparent (for example, there is no difference in QALY estimates in terms of whether the patient was recruited in a public or private hospital). Most notable is that generic QALYs and condition-specific QALYs have a similar ability to discriminate across patient and disease characteristics, reporting similar effect sizes.

Table 7 presents the results of a multivariate regression examining the differences in QALY estimates derived using the condition-specific measure (EORTC-8D) and the generic instrument (EQ-5D-3L). The average patient condition-specific QALYs are higher than generic QALYs, and the results in Table 7 suggest that this can be explained in part by the variation in baseline health state values, smoking status, changing ECOG status, advanced disease, death, and having prostate or bone and soft tissue cancer. A large variation in baseline health state values results in a greater difference in QALYs gained. In terms of clinically relevant variables, patients with prostate cancer (and marginally for those with bone and soft tissue cancer) relative to breast cancer patients have greater differences in terms of condition-specific and generic QALYs. Similarly those who experienced a decline in their ECOG performance relative to those who did not change performance status from baseline to their last follow-up point also have larger differences in QALY estimates, while those identified at baseline as having advanced disease thus requiring earlier follow-up and those who died during the course of follow-up led to smaller differences between the condition-specific QALY and the generic QALY.

**Table 7:** Regression results examining the difference in QALYs

	Coefficient	*p* value
Difference in baseline health state value	0.332	<0.001
Female	0.004	0.779
Age (reference <30 years)		
Aged 30–50 years	0.035	0.419
Aged 50–70 years	0.034	0.434
Aged >70 years	0.012	0.786
Private hospital recruitment	−0.027	0.107
Has hospital insurance	0.003	0.802
Smoking status (reference smoker)		
Ex-smoker	−0.039	0.011
Never smoked	−0.021	0.181
ECOG status (reference normal)		
Limited in normal activity	0.027	0.075
Self-care capable but not working	0.013	0.571
Limited self-care	−0.023	0.541
Change in ECOG status (reference no change)		
Decline in ECOG status	0.055	<0.001
Improvement in ECOG status	−0.013	0.527
Treatment intent (reference no treatment plan)		
Curative	−0.016	0.480
Palliative	−0.014	0.582
Planned three month follow-up (e.g. advanced disease)	−0.045	0.008
Dead at follow-up	−0.066	0.001
Site (reference breast)		
Prostate	0.046	0.045
Head and neck	0.038	0.102
Colorectal	−0.030	0.169
Lung	0.002	0.920
Bone and soft tissue	0.057	0.051
Cervical	0.013	0.690
CUP	−0.015	0.644
Renal	0.014	0.730
Oesophagogastric	0.009	0.762
All other	0.007	0.744
Stage (reference stage 1)		
Stage 0	0.059	0.309
Stage 2	−0.028	0.080
Stage 3	−0.003	0.883
Stage 4	0.003	0.870
Stage—other	0.030	0.307
Stage—not staged	−0.025	0.252
Constant	−0.023	0.711
Adjusted *R* ^2^	0.154	
*N*	1115	

Dependent variable is condition-specific QALYs minus generic QALYs

## Discussion

The health economics discipline has been debating condition-specific measures in the literature for a number of years [3, 5, 33, 34]. Recently there has been a plethora of condition-specific measures developed [35–39], but their use in decision-making remains limited [14]. This paper further informs the debate by testing the validity, responsiveness and sensitivity of a CSPBM for cancer. The EORTC-8D has previously been found to be broadly comparable to the EQ-5D [40], but that was within the same dataset that the EORTC-8D was developed from; thus, wider evaluation is required. This study provides one of the first external assessments of the instrument in comparison with EQ-5D-3L (see Hatswell et al. [41] for a comparison of EORTC-8D to SF-6D).

Descriptive analysis found that the mean health state value for the EORTC-8D was higher than for the EQ-5D-3L. Lloyd et al. [42] also found that the EORTC-8D scores were higher than EQ-5D-5L scores (a newer 5-level version of the EQ-5D [43]) in a group of men with metastatic castration-resistant prostate cancer. This may be a function of the EORTC-8D having a higher ‘floor’, and the lowest possible health state value is 0.292 compared with the EQ-5D-3L floor of −0.594. The greater range of values for the EQ-5D-3L may be observed because there is a greater opportunity for there to be lower values due to the theoretically plausible wider range of values that are available. Note that in the provisional EQ-5D-5L tariff for England the minimum value for the worst health state (55555) is −0.281 [44], while in other countries the worst health state values range from −0.446 [45] to −0.148 [46]. Further comparative analysis should be undertaken to consider the effect of the 5-level version, and note that Lloyd et al. [42] used a crosswalk from the 3L to the 5L. The EQ-5D-5L has been included in the next phase of Cancer 2015 [16].

Our assessment of convergent validity found that the dimensions and instruments were strongly correlated, while the analysis of content validity found few ceiling effects. Despite this, the agreement between the instruments was poor, with considerable variation in values for those with lower baseline HRQoL. Similar wide confidence intervals have been reported by others when comparing alternative generic MAUIs [47, 48].

The condition-specific QALYs estimated using the EORTC-8D were significantly higher than those derived from differences in the EQ-5D-3L over time (although the difference was small). Both the generic and condition-specific QALYs were found to be similarly sensitive to a number of patient and disease characteristics. Multivariate regression analysis of the difference in QALY estimates at a patient level found variation in baseline health state values had a large influence on the difference in QALYs gained, such that higher baseline EORTC-8D health state values relative to EQ-5D-3L values yield higher condition-specific QALYs compared to generic QALYs. This is despite the fact that higher baseline values mean there is less *utility space* in which to improve, given that health state values are bounded at 1. However, this may be driven by greater variation at the lower end of the health state values which would reaffirm the Bland–Altman results presented earlier (Fig. 2) where the poor agreement was driven by the patients with lower (mean) baseline HRQoL who also happened to have larger baseline differences.

Previous analysis [40] suggests that the EORTC-8D produced outcome values that are as valid, responsive and sensitive as EQ-5D-3L values. Our findings align with this, and at baseline the EQ-5D-3L and EORTC-8D values have a similar ability to discriminate between groups. This is also carried through to the QALY estimation where both generic and condition-specific QALYs appear equally responsive and sensitive to disease characteristics. When specifically considering the difference in QALYs, we find that this is most sensitive to differences in baseline health state values which are larger for those with lower HRQoL and in patients with declining performance and for particular sites. Therefore, researchers producing QALYs estimates from the EORTC-8D and decision makers utilising this information are advised to be cautious if their target group includes such patients. Caution is also advised if researchers/decision makers are using the instruments interchangeably (as may be the case in modelled economic evaluations) as the health state values differ considerably between instruments.

A limitation of this study is that while the cohort is rich in information it is not a clinical trial, and therefore, treatment effects vary. More research is required to compare the generic PBM and CSPBM in a trial setting. A further future limitation is that an additional PBM using the EORTC QLQ-C30 is underdevelopment (QLU-C10D) [49–51]. While the EORTC-8D classification system was derived using data from multiple myeloma patients, the new measure utilises data from multiple countries and multiple types of cancer to derive its classification system and in addition aims to produce country-specific preference weights for a range of different countries including the UK and Australia. Both instruments draw on the EORTC QLQ-C30 which the EORTC Quality of Life Group suggests is supplemented by disease-specific modules (e.g. QLQ-BR23 for breast, QLQ-MY20 for multiple myeloma). Therefore, it may be that more specificity is required with oncology assessments and both of these CSPBM require further supplementation.

There is growing concern with respect to the high cost of personalised or targeted drugs for cancer treatment [52, 53]; the greater financial risk means that it is even more important to accurately measure the outcomes of treatment to estimate if treatment offers value for money. Our research suggests that CSPBMs offer both similarities and differences to generic PBMs, and while this difference equates to marginally higher QALYs in our cohort, further research is required to confirm if these higher QALYs offer a more accurate reflection of HRQoL gains [54].

## Appendix

See Tables 8 and 9.

**Table 8:** EORTC-8D responses to each dimension (% of respondents)

	Level 1	Level 2	Level 3	Level 4	Level 5
Physical functioning	51.43	23.84	13.41	9.42	1.91
Role functioning	55.18	21.93	12.63	10.25	n/a
Pain	59.59	24.91	9.24	6.26	n/a
Emotional functioning	58.40	29.86	8.34	3.40	n/a
Social functioning	53.81	24.91	13.59	7.69	n/a
Fatigue and sleep disturbance	30.87	45.47	17.10	6.56	n/a
Nausea	77.83	15.02	5.01	2.15	n/a
Constipation/diarrhoea	59.30	27.59	8.22	4.89	n/a

**Table 9:** EQ-5D-3L responses to each dimension (% of respondents)

	Level 1	Level 2	Level 3
Mobility	77.29	22.11	0.60
Self-care	91.60	7.57	0.83
Usual activities	61.86	30.33	7.81
Pain/discomfort	48.33	46.90	4.77
Anxiety/depression	56.85	39.15	3.99
